# Assessment of Sperm Viability and Computer-Assisted Motility Analysis in Budgerigars (*Melopsittacus undulatus*): Effect of Several *In Vitro* Processing Conditions

**DOI:** 10.1155/2022/5997320

**Published:** 2022-03-22

**Authors:** Manuela Madeddu, Stefano Marelli, Ahmad Abdel Sayed, Fabio Mosca, Silvia Cerolini, Luisa Zaniboni

**Affiliations:** Department of Veterinary Medicine and Animal Sciences, University of Milan, via dell'Università 6, Lodi 26900, Italy

## Abstract

In order to preserve endangered psittacine species, more basic and applied research in reproductive biology is required. Assisted reproductive technologies such as artificial insemination play an important role in parrots species conservation programs to overcome the problem of infertile eggs and male infertility. The aim of this study was to define an effective *in vitro* protocol in order to standardize the sperm quality evaluation in psittacines, studying *Melopsittacus undulatus* as model species. Semen was collected from twenty adult males by massage technique from May to June. Sperm concentration was measured by the spectrophotometric method. Sperm quality (sperm membrane integrity (SMI), motility, and kinetic parameters) was assessed on fresh semen. Three different experimental protocols were performed to compare the effects of various processing conditions on SMI, motility, and kinetic parameters. In protocol 1, test was performed by Lake extender with three different pH, 7.4 *versus* 8.2 *versus* 8.4, and two different equilibration temperatures after dilution of fresh semen (4°C *versus* 25°C). In protocol 2, two dilution rates of semen after collection were valuated, 1 : 3 *versus* 1 : 4, as well as three different semen storage temperatures (4°C *versus* 25°C *versus* 38°C) before sperm motility analysis with the computer-assisted sperm analysis (CASA). In protocol 3, two different Makler chamber temperatures (38 *versus* 41°C) during motility analysis were tested. A significant progressive improvement in spermatozoa motility and kinetic parameters was registered with pH 8.4. Progressive motility and all kinetic parameters were higher at 4°C equilibration temperature. Straightness (STR) kinetic parameter was better with 1 : 4 dilution rate. Total motile sperm was higher in 41°C Makler chamber. In this study, for the first time, the effects of different processing protocols on psittacines seminal quality analysis were investigated. Significant differences conditioning the effectiveness of analysis protocols have been described.

## 1. Introduction

Artificial insemination (AI) is an assisted breeding technique described in birds by Quinn and Burrows [1] and widely studied in domesticated species, firstly in chickens and also in turkeys, ducks, and geese [2, 3]. The poultry industry took advantage of this breeding technique for commercial purposes and nowadays relies on the use of AI for breeding commercial strain lines in turkey populations.

AI has been also successfully transferred to a variety of wild birds, including raptors, psittacines, and passerines species [4]. AI efforts in wild bird species have mainly been research-oriented to evaluate its applicability [5]. However, AI can play an important role in conservation and reintroduction programs improving the reproductive efficiency in *ex situ in vivo* conservation nuclei [6]. The combination of *in situ* and *ex situ* conservation techniques has been analysed and has been described as an objective model showing assisted breeding efficacy in dangered species recovery [5].

According to the International Union for Conservation of Nature Red List, 13% of birds are threatened with extinction, and the same proportion increases to 28% in psittacine order [7]. Understanding and improving reproduction are essential ingredients in the avian biodiversity conservation [6].

Semen collection and its quality assessment are the first fundamental actions to identify good semen donors in order to perform successful AI. In psittacines, two semen collection methods have been described in literature according to the size of the birds. The abdominal massage method, firstly studied in chicken [8], was used in small psittacine species with species-specific adaptation [9, 10] providing 74% of successful semen collections in cockatiels [11]. However, this noninvasive method requires frequent handling of birds and a training period to get them used to stimulation and milking. Electrostimulation was suggested as novel method to collect semen in large parrots providing 67% of successful attempts [12]. However, when it was applied to Spix's Macaws (*Cyanopsitta spixii,* very rare and endangered species probably surviving only in captivity), the success rate was decreased to 44% [13]. In a study comparing different orders of birds, semen collection by electrostimulation was largely variable according to order, species, and season. In psittacines, the overall success rate was quite low, being 19% in Blu-fronted amazon (*Amazona aestiva*), 15% in Hyacinth macaw (*Anodorhynchus hyacinthinus*), and 25% in Blue-winged macaw (*Primolius maracana*) [14]. The choice of the method for semen collection in psittacines is still an open question and is based on the advantage/disadvantage balance related to species, seasonality, housing, and available equipment.

The assessment of semen quality is usually based on standard parameters, including sperm concentration, viability, and motility, and objective procedures were studied in fowl semen [15]. Motility is one of the most important sperm characteristics and it is widely accepted that it is associated with fertilization. In birds, sperm motility assessed by objective procedures was recognised as a predictor of male fertility and suggested to select male sires in both chickens and turkeys [16, 17]. Computer-assisted semen analysis (CASA) was developed in 1980s to provide an objective method to assess sperm motility and kinetic parameters and is nowadays routinely used in mammals and birds [18–22]. About psittacine, CASA has been used to characterise the quality of fresh semen in cockatiels (*Nymphicus hollandicus*) [23] and Rosy-faced lovebirds (*Agapornis roseicollis*) [24] and also in budgerigars cryopreserved semen (*Melopsittacus undulatus*) [25, 26]. However, CASA provides highly variable results dependent on *in vitro* semen processing conditions; therefore analysis process and quality control procedure definition play a pivotal role in CASA report preparation [19].

No data are available on the effect of semen processing conditions for the assessment of semen quality in psittacines. Therefore, the aim of the present study was to identify the best *in vitro* processing condition in order to standardize the assessment of sperm membrane integrity (SMI) by fluorescent staining, sperm motility, and kinetic parameters by CASA in semen samples collected by the abdominal massage technique from budgerigars, used as model within psittacines species. The success rate of sperm collection attempts using a modified massage technique was also evaluated.

## 2. Materials and Methods

### 2.1. Bird Management and Semen Collection

Twenty adult *Melopsittacus undulates* males, 1-2 years old, belonging to a private aviary (Monza, Italy) were sampled. Birds were housed in standard cages (size 180 × 60 × 60 cm), divided in four groups (4 males + 1 female/cage), kept at 20°C with 14 hours of light: 10 hours of dark as photoperiod within a dedicated shelter. The budgerigars were *ad libitum* fed a standard commercial seed mixture and drinking water.

Bird handling was in accordance with the principles presented in Guidelines for the Care and Use of Agricultural Animals in Research and Teaching [27]. After 2-week training period for handling and semen collection, all males were routinely collected twice a week for two months (May-June). Before semen collection, each male was fasted for at least 1 hour and transferred to a clean cage; semen collection started after the first faecal ejection to minimize ejaculate contamination. Semen was collected according to the Burrows and Quinn massage technique [8] with few modifications due to the budgerigars' specific size and behaviour. Briefly, the birds were kept flat on their back in one hand and the legs were firmly kept with the thumb finger; a paper tube was positioned around the head and the wings. Spontaneous ejaculation was obtained after ventral abdominal massage practiced by the operator holding the bird. If no spontaneous ejaculation occurred, semen could yet be milked out of the deferent duct by a gentle squeezing of the cloaca. Urine or faecal contaminated ejaculates were discarded. The ejaculates were collected into graduated glass microtubes and volumes were recorded.

Each day of collection, males were divided into 4 different groups (4 birds/group) and all ejaculates collected within one group were pooled into one semen sample. Males within group were changed every collection time to minimize the bird effect.

### 2.2. Sperm Concentration Assessment

Soon after collection, ejaculates were pooled, diluted in Lake extender (LE) [28], and equilibrated for 30 min to allow the transfer to the laboratory for sperm quality analyses (see below in “Experimental Protocol 1”).

Sperm concentration (SC) was measured by the spectrophotometric method [15]. Firstly, a linear standard regression equation was studied using serial dilutions in 0.9% NaCl of neat semen. The concentration range considered was 0.825 × 10^9^–9.85 × 10^9^ sperm/mL and the final linear standard regression equation was as follows: sperm concentration = 18,01 ^*∗*^ (optical density, OD)-0,463, *R*^2^ = 0.99%. After equation setting, OD was recorded in semen samples diluted 1 : 400 in 0.9% NaCl by a calibrated photometer (IMV, L'Aigle, France) at a wavelength of 535 nm. Then, OD was converted into SC (×10^9^ sperm/mL) by the linear standard regression equation.

### 2.3. Assessment of Sperm Viability and Motility

SMI was assessed by dual SYBR14/propidium iodide fluorescent staining procedure already used for chicken semen [20]. The LIVE-DEAD Sperm Viability Kit (Life Technologies, Italy) was used. In brief, semen samples were diluted to 100 × 10^6^/mL in LE; 5 *μ*L of diluted semen was added to 44 *μ*L of LE containing 1 *μ*L SYBR14 (diluted 1 : 100 in dimethyl sulfoxide) and was incubated for 10 min at 25°C. Then, 5 *μ*L propidium iodide (diluted 1 : 100 in LE) was added and further incubated for 5 min at 25°C. The assessment of 200 spermatozoa was performed in duplicate aliquots at 1000x total magnification using a fluorescent microscope (Axioskop 40- AxioCam ICc 1, Zeiss) and FITC filter fluorescence. The proportion of sperm with undamaged sperm membrane was calculated with the following formula: number of green sperms × 100/total number of sperms.

Sperm motility and kinetic parameters were assayed using a CASA system coupled to a phase contrast microscope (Nikon Eclipse model 50i; negative contrast) employing the Sperm Class Analyzer (SCA) software (version 4.0, MICROPTIC S.L., Barcelona, Spain). Semen was diluted in 0.9% NaCl to a sperm working concentration (SWC) of 100 × 10^6^/ml and 10 *μ*l were placed on a Makler counting chamber (Sefi Medical Instruments, Haifa, Israel) and evaluated under the microscope. A minimum of 3 fields and 500 sperm tracks were analysed at 100x total magnification; 25 frames per second (Hz) and 25 frames per field were set. The following software settings were used: range cell size was from 5 to 190 *μ*m^2^; sperm was classified as motile if VCL ≥13  m/s; sperm was classified as rapid if VCL >100  m/s, medium if 100  m/s > VCL >50  m/s, slow if 50 *μ*m/s > VCL >13  m/s, and static if VCL< 13  m/s; sperm was classified as progressive if STR ≥70%. The following SCA parameters were recorded: total motile sperm (TMS, %), progressive motile sperm (PMS, %), curvilinear velocity (VCL, (*μ*m/s)), straight-line velocity (VSL, (*μ*m/s)), average path velocity (VAP, (*μ*m/s)), amplitude of lateral head displacement (ALH, (*μ*m)), and beat cross frequency (BCF, (Hz)). The following velocity ratios were also recorded: linearity of the curvilinear path (LIN = VSL/VCL), straightness of the average path (STR = VSL/VAL), and wobble of the actual path about the average path (WOB = VAP/VCL) [19].

### 2.4. Experimental Protocols

The effects of several *in vitro* processing conditions on the assessment of SMI, TMS, PMS, and kinetic parameters were studied in consecutive experimental protocols. In each experimental protocol, at least 7 pooled semen samples, corresponding to 7 replicates/treatment, were processed according to the experimental conditions.

#### 2.4.1. Experimental Protocol 1

A factorial design was used to study the following treatments: 1a) LE pH: 7.4 *versus* 8.2 *versus* 8.4; 1b) equilibration temperature (ET) after dilution of fresh semen: 4°C *versus* 25°C. Ejaculates were pooled in semen samples, each one divided in 3 aliquots diluted 1 : 3 in LE with different pH, and each diluted sample was split into two aliquots, one kept at 4°C and the other at 25°C for 30 min, during transport to the laboratory. SMI, TMS, PMS, and kinetic parameters were assayed as described above; as concerning CASA analysis, dilution to SWC in saline solution was performed at room temperature for 1 min and Makler chamber was preheated to 38°C.

#### 2.4.2. Experimental Protocol 2

A factorial design was used to study the following treatments: 2(a) dilution rate (vol:vol) in 8.4 pH LE of neat semen soon after collection: 1 : 3 *versus* 1 : 4; 2(b) temperature of 0.9% NaCl for dilution to SWC before SCA assessment: 4°C *versus* 25°C *versus* 38°C. Ejaculates were pooled in semen samples, each one divided in 2 aliquots diluted 1 : 3 and 1 : 4 in 8.4 pH LE and kept at 4°C for 30 min. SMI was assayed as described above; SCA analysis was performed after further semen dilution to SWC in saline solution for 1 min according to treatment (2b) and preheating the Makler chamber to 38°C.

#### 2.4.3. Experimental Protocol 3

The treatment studied was the temperature of the Makler chamber during SCA analysis: 38°C *versus* 41°C. Ejaculates were pooled in semen samples, diluted 1 : 4 in 8.4 pH LE, and kept at 4°C for 30 min. SMI was assayed as described above; SCA analysis was performed after further semen dilution to SWC in 25°C saline solution for 1 min and preheating the Makler chamber to 38°C and 41°C.

### 2.5. Statistical Analyses

Analysis of variance on sperm variables was performed using the GLM procedure of SAS [29]. The sources of variation included in the statistical model are reported according to the experimental protocol: experimental protocol 1 included pH of LE, ET after dilution, and the relative interaction; experimental protocol 2 included dilution of neat semen, temperature of 0.9% NaCl for dilution to SWC before SCA analysis, and relative interaction; experimental protocol 3 included the Makler chamber prewarming temperature.

*Student's t*-test was used to compare LSMeans, and statistical significance was set at *P* < 0.05. Prior to statistical analyses, all percentage data were normalized with an arcsine transformation. Results are presented as LSMean ± SE.

## 3. Results

Birds have been easily trained to the handling for semen collection and the majority of birds produced semen on each day of collection. The mean daily proportion of semen donor birds was 74% over the experimental period and it ranged from 58% to 89% according to the day of collection. The ejaculates of budgerigars showed peculiar characteristics and descriptive statistics to describe semen volume and concentration are reported in Table 1. Ejaculate volume was very low, just few microliters, and was balanced by a high sperm concentration, with the average value being 8 × 10^9^/mL; large variability was found in both measures between birds and collections; therefore, high coefficients of variations (CVs) were calculated.

### 3.1. Experimental Protocol 1

The results of the analysis of variance are reported in Table 2. The pH of the extender and the ET of diluted semen significantly affected (*P* < 0.001) the majority of the sperm motion variables and, in contrast, did not significantly affect SMI; the relative interaction pH *∗* ET did not show any significant effect (Table 2).

The mean values for SMI, TMS, PMS, and kinetic parameters recorded in semen diluted with LE at different pH are reported in Table 3. A significant progressive improvement according to the progressive increase of the pH value was observed in the proportion of motile and progressive sperm and in the kinetic parameters VCL, VSL, VAP, LIN, ALH, WOB, and BCF.

SMI, TMS, PMS, and kinetic parameter mean values recorded in semen samples kept at different ET after dilution are reported in Table 4. Again, SMI did not significantly change between samples kept at 4°C and 25°C, whereas significantly higher mean values were recorded in TMS and all kinetic parameters when semen samples were kept at 4°C (Table 4).

### 3.2. Experimental Protocol 2

In experimental protocol 2, the best processing conditions worked out in protocol 1 were used. Therefore, semen samples were diluted in 8.4 pH LE and kept at 4°C for 30 min before further semen quality analyses.

According to the results of analysis of variance, the dilution rate of neat semen significantly (*P* < 0.05) affected only the kinetic parameters STR and the mean values recorded in the experimental groups are reported in Table 5. In contrast, the temperature of 0.9% NaCl to dilute semen to the SWC before SCA assay and the relative interaction did not significantly affect sperm motion variables (*P* > 0.05). However, despite no significant effect, a general negative trend was observed in sperm motion parameters increasing the 0.9% NaCl temperature from 25°C to 38°C (data not shown). Therefore, the temperatures to dilute budgerigar semen to the SWC before CASA analysis in the range of 4°C–25°C and 4°C are suggested to keep a constant temperature until data acquisition.

### 3.3. Experimental Protocol 3

Analysis of variance showed that preheating of the Makler chamber to the temperatures of 38 and 41°C significantly affected only the proportion of TMS (*P* < 0.05) and in contrast no significant effect was found on PMS proportion and kinetic parameters. The mean proportion of TMS was more than doubled increasing the temperature of the Makler chamber from 38 to 41°C, being 16.2% and 36.3%, respectively (*P* < 0.05).

## 4. Discussion

Many psittacine species are endangered because of loss of their natural habitat and poaching, but since research potentiality on wild birds is restricted, common species have been proposed as a model to intensify the knowledge in sperm collection and assessment [11, 24–26]. The cloacal massage technique was chosen for sperm collection to take advantage of its benefits, such as short training period, being adapted to many different species and noninvasive [5]. The present study confirms that the abdominal massage method routinely used for semen collection in domestic fowl [8] can be successfully used to stimulate ejaculation in the budgerigar (*Melopsittacus undulates*), presumably the parrot species most usually bred in captivity. Parrots were easily trained to the manipulation modified according to their size and behaviour; ejaculation of semen was successful in 74% of attempts, in agreement with the rates obtained in other wild birds, such as cockatiels (74,2%) [11], the Indian white-backed vulture (84.6%) [30], the American kestrel (74%) [31], the sandhill crane (86%) [32], and the blue rock pigeon (90%) [33], using the same method.

Semen quality assessment includes quantitative and qualitative parameters. The quantitative parameters, semen volume and concentration, have been assessed by objective standardized techniques and the mean values described in this study were similar and even higher compared with data reported in *Melopsittacus undulatus* bred in promiscuous aviary or in couple [25, 26].

Among qualitative parameters, the most widely used in the standard semen assessment are sperm viability, morphology, and motility [34]. The small ejaculate volume of budgerigars limits the assessment of sperm quality to few parameters; therefore it is essential to standardize the sample evaluation techniques to collect objective and reliable data.

The dual staining SYBR14/PI fluorescent technique used to assess SMI in different poultry species [20, 35] has been successfully transferred to budgerigar semen and it was not affected by the *in vitro* processing conditions tested in the experimental protocols. SMI mean values reported in this study range from 86 to 88% and are higher compared with the values assessed with the same technique in budgerigar males housed in a promiscuous aviary with female and in couple [25]. No other data are available in the literature reporting sperm viability in *Melopsittacus undulatus*. In cockatiels (*Nymphicus hollandicus*), sperm viability was reported to range from 90 to 93.3% [23], in agreement with the present data.

The assessment of sperm motility, progressive motility, and kinetic parameters performed with the SCA software was affected by the *in vitro* processing conditions, corresponding to pH of the extender, dilution rate, equilibration temperature, and Makler temperature for data acquisition.

The pH of the semen extender must be adequate to achieve success in artificial insemination, unless insemination occurs within a short time from semen collection. In nondomestic birds, if there is no species-specific information, extenders for poultry species are commonly used, with a pH of around 7, but they may not give optimal results [9]. Samour et al. [36] found that sperm motility and insemination success in budgerigars improved when using a modified extender with pH 8.3 compared to a standard poultry extender. In the present study, motility values and kinetic parameters were progressively improved with the progressive increase of the extender pH from 7.8 to 8.4. A similar positive effect of alkaline pH on the proportion of motile sperm and the motion velocity was previously found in chicken, turkey, and quail semen; furthermore, pH effect on sperm motility was affected by temperature and more alkaline conditions were required to stimulate motility at 40°C compared to 30°C [37]. A positive effect of the combination of alkaline pH and high temperature (40°C) on sperm motility and kinetic parameters was found also in ostrich semen [38]. The best status of the spermatic movements was observed at pH 7.5 and 8 in the Tree Sparrow as well [39]. A pH dependent effect on sperm motility was hypothesized to be present in the female reproductive tract, being functional to promote sperm quiescence within the SST (sperm storage tubules) and sperm activation before fertilization [37].

Semen extender is highly important for the use of both fresh and stored semen to maintain the viability of sperm *in vitro*. The extender provides substrates for sperm metabolism and prevents the toxic effects of its products reducing the risk of sperm deterioration during *in vitro* storage. Semen extension is also important since poultry semen is very viscous and concentrated and the appropriate dilution allows maximizing the number of females potentially inseminated [40]. It is well known that the “dilution effect” concerns a dramatic decline in sperm motility caused by excessive semen dilution [41]. In this study, the increased dilution of semen positively influenced only STR, one of the essential markers of progressive motility. A positive effect of dilution in stallion sperm was described in the work of Varner et al. [42]. In their study, total sperm motility and progressive sperm motility were higher at 25 than at 50 or 100 × 10^6^ sperm/mL after 12 hours of storage in a skim milk-glucose extender.

In domestic poultry, spermatozoa suspended in a saline solution increase their motility when the temperature rises from 20 to 35°C, while above this temperature sperm motility decreases until they become completely immobile at avian body temperature (40–41°C) [43]. This phenomenon is reversible, as Munro [44] first reported, and spermatozoa regain motility when the temperature is cooled to 30°C. In this study, TMS and kinetic parameters showed better values when equilibration temperature was at 5°C *versus* 25°C, while TMS was higher when temperature of the Makler chamber was at 41°C *versus* 38°C. In the Blue rock pigeon, a positive effect of high temperature (24 and 37°C) on sperm motility was found during 7 hours of *in vitro* semen storage compared to low temperature (4°C) that provided no motile sperm after 4-hour storage [33]. Gloria et al. [25] and Dogliero et al. [26] registered higher motility values in budgerigar semen kept at 35°C within 10 min after collection and 37,5°C within 5 min after collection, respectively, using a different CASA equipment. The comparison between results is difficult because these differences may depend on many factors related to semen processing, such as extender type, dilution rate and interval time between collection and analysis, and CASA settings. In this work, the analyses were carried out after 30 minutes from collection and the best values obtained at 4°C *versus* 25°C can be related to the reduction of the cellular metabolic activity and microbial growth during cooling [45]. The high temperature of the Makler chamber reactivates cell activity causing a sperm motility recovery. To preserve fertilization capacity in poultry, pooled semen is normally stored at 2–8°C so that sperm metabolism decreases and less reactive oxygen species (ROS) are produced [46–48]. In the stallion, a temperature about 5°C has been determined as the optimal storage temperature for maintenance of motility and fertility [45]. Yang et al. [39] evaluated sperm motility in the Tree Sparrow semen and, after 15 minutes of incubation at 40°C with a pH of 7.5, no more progressive sperms were detected. The CASA tool differs mainly between machines, based on software and optic used for sperm identification and trajectory rebuilding. The authors used Hamilton Thorne and set 60 frames per second (Hz) while in this study Sperm Class Analyzer (SCA, Microptic) was used and 25 frames per second (Hz) were set. Considering the high heterogeneity of sperm motility between species, it is essential that the CASA settings must be standardized according to the species [49] and the results of computerized semen analysis cannot be compared among various laboratories if identical parameter settings are not used [50].

## 5. Conclusions

Species conservation with reproductive biology should be systematic, with basic and applied research, in order to improve breeding programs and to develop assisted reproductive techniques for endangered species. Therefore, it is essential to establish standard procedures for semen laboratory analyses in each species according to their peculiar reproductive biology. For the first time, the effect of the *in vitro* processing conditions on sperm quality assessment was studied in budgerigar semen using objective techniques. According to the present results, it is recommended to dilute the *Melopsittacus undulates* semen sample 1 : 4 in 8.4 pH extender soon after collection and then to equilibrate at 4°C for 30 minutes. SMI can be assessed using the dual staining SYBR14/PI standard technique already used for chicken semen, whereas the assessment of sperm motility and kinetic parameters by SCA software requires the following steps before data recording: dilution to 100 × 10^6^ sperm/mL in saline solution at 4°C and then loading into a Makler chamber prewarmed to 41°C. In endangered species, AI is the reproductive technique allowing successful breeding in captive populations and is specifically useful to overcame breeding failures in birds with physical or behavioural disabilities in order to guarantee and conserve genetic variability in the new generations.

## Figures and Tables

**Table 1:** Descriptive statistics of semen quality parameters recorded in budgerigars.

Semen parameters	Mean	SD^1^	SE^2^	Min	Max	CV^3^
Volume (*μ*L)	2.6	1.0	0.2	1.0	4.5	39.2
Concentration (×10^9^/mL)	8.2	2.6	0.6	4.0	13.6	32.2

^1^Standard deviation. ^2^Standard error. ^3^Coefficient of variation.

**Table 2:** Results of analysis of variance: significance (*P*) of the sources of variation affecting sperm viability and CASA parameters in experiment 1.

Sperm variables^1^	Sources of variation^2^		
	pH	ET	pH^*∗*^ET
SMI (%)	ns	ns	ns
TMS (%)	0.0004	0.0196	ns
PMS (%)	0.0096	ns	ns
VCL (*μ*m/s)	0.0003	0.0175	ns
VSL (*μ*m/s)	0.0001	0.0001	ns
VAP (*μ*m/s)	0.0001	0.0004	ns
LIN (%)	0.0003	0.0001	ns
STR (%)	ns	0.0001	ns
WOB (%)	0.0001	0.0003	ns
ALH (*μ*m)	0.0007	0.0001	ns
BCF (Hz)	0.0203	0.0001	ns

^1^SMI: sperm membrane integrity; TMS: total motile sperm; PMS: progressive motile sperm; VCL: curvilinear velocity; VSL: straight-line velocity; VAP: average path velocity; LIN = VSL/VCL × 100; STR = VSL/VAP × 100; WOB = VAP/VCL × 100; ALH: amplitude of lateral head displacement; BCF: beat cross frequency. ^2^pH = pH of Lake extender; ET: equilibration temperature after semen dilution.

**Table 3:** Sperm viability and CASA parameters (LSMeans ± SE) measured in budgerigar semen samples diluted in Lake extender (LE) at different pH.

Sperm variables^1^	pH		
	7.8	8.2	8.4
SMI (%)	86.3 ± 1.4	86.8 ± 1.1	87.5 ± 1.9
TMS (%)	6.0 ± 3.0^a^	15.5 ± 2.4^b^	26.2 ± 3.8^c^
PMS (%)	0.3 ± 0.6^a^	1.0 ± 0.4^a^	3.0 ± 0.7^b^
VCL (*μ*m/s)	21.6 ± 1.0^a^	25.2 ± 0.8^b^	28.3 ± 1.3^c^
VSL (*μ*m/s)	5.7 ± 0.9^a^	8.7 ± 0.7^b^	12.6 ± 1.2^c^
VAP (*μ*m/s)	10.3 ± 1.0^a^	14.3 ± 0.8^b^	18.4 ± 1.3^c^
LIN (%)	25.0 ± 2.4^a^	33.4 ± 1.9^b^	41.3 ± 3.0^c^
STR (%)	55.9 ± 2.4	57.9 ± 1.9	61.5 ± 3.0
WOB (%)	44.9 ± 2.4^a^	55.3 ± 1.9^b^	62.7 ± 3.0^c^
ALH (*μ*m)	0.5 ± 1.2^a^	1.2 ± 0.1^b^	1.5 ± 0.2^b^
BCF (Hz)	1.5 ± 0.5^a^	2.9 ± 0.4^b^	3.9 ± 0.7^b^

^1^SMI: sperm membrane integrity; TMS: total motile sperm; PMS: progressive motile sperm; VCL: curvilinear velocity; VSL: straight-line velocity; VAP: average path velocity; LIN = VSL/VCL × 100; STR = VSL/VAP × 100; WOB = VAP/VCL × 100; ALH: amplitude of lateral head displacement; BCF: beat cross frequency. ^a,b,c^Different superscripts show a significant difference between treatments within row at *P* < 0.05.

**Table 4:** Sperm viability and CASA parameters (LSMeans ± SE) measured in budgerigar semen samples diluted in Lake extender (LE) and equilibrated at 25°C and 4°C.

Sperm variables^1^	ET^2^	
	25°C	4°C
SMI (%)	86.6 ± 1.5	87.1 ± 0.9
TMS (%)	11.3 ± 3.3^a^	20.5 ± 1.9^b^
PMS (%)	0.9 ± 0.69	1.9 ± 0.36
VCL (*μ*m/s)	23.5 ± 1.1^a^	26.6 ± 0.6^b^
VSL (*μ*m/s)	6.6 ± 1.0^a^	11.4 ± 0.6^b^
VAP (*μ*m/s)	11.9 ± 1.19^a^	16.7 ± 0.6^b^
LIN (%)	26.2 ± 2.6^a^	40.3 ± 1.5^b^
STR (%)	50.8 ± 2.6^a^	66.1 ± 1.5^b^
WOB (%)	44.6 ± 2.6^a^	60.0 ± 1.5^b^
ALH (*μ*m)	0.5 ± 1.2^a^	1.6 ± 0.1^b^
BCF (Hz)	0.9 ± 0.6^a^	4.7 ± 0.3^b^

^1^SMI: sperm membrane integrity; TMS: total motile sperm; PMS: progressive motile sperm; VCL: curvilinear velocity; VSL: straight-line velocity; VAP: average path velocity; LIN = VSL/VCL × 100; STR=VSL/VAP × 100; WOB=VAP/VCL × 100; ALH: amplitude of lateral head displacement; BCF: beat cross frequency. ^2^Equilibration temperature. ^a,b^Different superscripts show a significant difference between treatments within row at *P* < 0.05.

**Table 5:** Sperm viability and CASA parameters (LSMeans ± SE) measured in budgerigar semen samples diluted in 8.4 pH Lake extender (LE) at different rate before quality assessment.

Sperm variables^1^	Dilution rate	
	1 : 3	1 : 4
SMI (%)	87.7 ± 1.3	86.5 ± 2.6
TMS (%)	29.9 ± 2.8	25.8 ± 5.3
PMS (%)	4.1 ± 0.6	3.5 ± 1.2
VCL (*μ*m/s)	31.3 ± 0.9	33.2 ± 1.7
VSL (*μ*m/s)	15.3 ± 1.0	18.7 ± 2.0
VAP (*μ*m/s)	21.4 ± 1.0	24.3 ± 2.0
LIN (%)	46.9 ± 2.0	55.2 ± 3.7
STR (%)	68.6 ± 1.5^a^	75.1 ± 2.8^b^
WOB (%)	66.8 ± 1.5	72.6 ± 2.9
ALH (*μ*m)	2.1 ± 0.1	2.3 ± 0.2
BCF (Hz)	5.9 ± 0.4	7.0 ± 0.7

^1^SMI: sperm membrane integrity; TMS: total motile sperm; PMS: progressive motile sperm; VCL: curvilinear velocity; VSL: straight-line velocity; VAP: average path velocity; LIN = VSL/VCL × 100; STR=VSL/VAP × 100; WOB=VAP/VCL × 100; ALH: amplitude of lateral head displacement; BCF: beat cross frequency. ^a,b^Different superscripts show a significant difference between treatments within row at *P* < 0.05.

## Data Availability

The data are provided in the Supplementary Materials.
