# Supplementary material for: Assessment of Sperm Viability and Computer-Assisted Motility Analysis in Budgerigars (Melopsittacus undulatus): Effect of Several In Vitro Processing Conditions
Source: Vet Med Int. 2022 Mar 22;2022:5997320. doi: 10.1155/2022/5997320 (PMC8964162; doi:10.1155/2022/5997320)
Supplement: Supplementary Materials — The data are provided in the Supplementary Materials. [file 5997320.f1.zip › 5997320.f1/dataset_protocollo_1_pappagalli.pdf]

pool	pH	TT	Vivi	Morti	TOT	Vitalità	Statici	li non prog
1	7	f	161	39	200	80.5	82.1	16.1
1	7	f	161	39	200	80.5	93.9	5.7
1	7	c	161	39	200	80.5	94.6	5.3
1	7	c	161	39	200	80.5	96.9	3.1
1	8	f	161	39	200	80.5	84.8	13.6
1	8	f	161	39	200	80.5	91.6	8.2
1	8	c	161	39	200	80.5	95.1	4.7
1	8	c	161	39	200	80.5	95.3	4.5
2	7	f	183	30	213	85.9	94.4	5.4
2	7	f	183	30	213	85.9	96.4	3.6
2	7	c	183	30	213	85.9	96.2	3.8
2	7	c	183	30	213	85.9	92.9	6.9
2	8	f	183	30	213	85.9	92.1	7.4
2	8	f	183	30	213	85.9	85.7	14.1
2	8	c	183	30	213	85.9	97.1	2.9
2	8	c	183	30	213	85.9	76.2	23.8
3	7	f	181	45	226	80.1	95.3	4.7
3	7	f	181	45	226	80.1	97.1	2.9
3	7	c	181	45	226	80.1	95.4	4.6
3	7	c	181	45	226	80.1	97.6	2.4
3	8	f	181	45	226	80.1	73.7	24.1
3	8	f	181	45	226	80.1	84.3	15
3	8	c	181	45	226	80.1	94.7	5.3
3	8	c	181	45	226	80.1	92.1	7.8
4	7	f	206	24	230	89.6	84.2	13.9
4	7	f	206	24	230	89.6	98.8	1.2
4	7	c	206	24	230	89.6	97	3
4	7	c	206	24	230	89.6	96.3	3.7
4	8	f	206	24	230	89.6	70	26.7
4	8	f	206	24	230	89.6	73.3	26.3
4	8	c	206	24	230	89.6	92.6	7.2
4	8	c	206	24	230	89.6	95.1	4.9
5	7	f	197	19	216	91.2	87.2	11.9
5	7	f	197	19	216	91.2	98.2	1.8
5	7	c	197	19	216	91.2	94.7	5.3
5	7	c	197	19	216	91.2	95.6	4.4
5	8	f	197	19	216	91.2	86.9	12.4
5	8	f	197	19	216	91.2	92.5	7.2
5	8	c	197	19	216	91.2	95	5
5	8	c	197	19	216	91.2	95.5	4.5
6	7	f	186	20	206	90.3	92	7.8
6	7	f	186	20	206	90.3	94.8	4.7
6	7	c	186	20	206	90.3	93.9	6.1
6	7	c	186	20	206	90.3	90.8	9.2
6	8	f	186	20	206	90.3	77.9	19.8
6	8	f	186	20	206	90.3	78.7	21.3
6	8	c	186	20	206	90.3	94.5	5.5
6	8	c	186	20	206	90.3	86.4	12.7
1	8.2	f	169	34	203	83.3	94.7	5.1

1	8.2	f	169	34	203	83.3	89.5	10
1	8.4	f	169	34	203	83.3	86.8	12.8
1	8.4	f	169	34	203	83.3	86.7	13.2
2	8.2	f	174	39	213	81.7	56.9	40.3
2	8.2	f	174	39	213	81.7	43.8	50.8
2	8.4	f	174	39	213	81.7	50.2	43.1
2	8.4	f	174	39	213	81.7	57	37.9
3	8.2	f	171	66	237	72.2	96.4	3.4
3	8.2	f	171	66	237	72.2	97	3
3	8.4	f	171	66	237	72.2	94.1	5.7
3	8.4	f	171	66	237	72.2	94.4	5.6
4	8.2	f	169	38	207	81.6	91.5	8.2
4	8.2	f	169	38	207	81.6	88.3	11.4
4	8.4	f	169	38	207	81.6	79.1	18.8
4	8.4	f	169	38	207	81.6	55.8	39.9
5	8.2	f	218	8	226	96.5	70.5	28.2
5	8.2	f	218	8	226	96.5	78.3	21.2
5	8.4	f	218	8	226	96.5	65.3	32.2
5	8.4	f	218	8	226	96.5	46	47.6
6	8.2	f	194	6	200	97.0	18.2	65.4
6	8.2	f	194	6	200	97.0	69.7	26.6
6	8.4	f	194	6	200	97.0	50.1	42.1
6	8.4	f	194	6	200	97.0	62	33.4
7	8.2	f	206	12	218	94.5	60.3	34.9
7	8.2	f	206	12	218	94.5	50.6	47.2
7	8.4	f	206	12	218	94.5	34.9	47.7
7	8.4	f	206	12	218	94.5	40.4	56
8	8.2	f	206	14	220	93.6	93.6	6.4
8	8.2	f	206	14	220	93.6	93.3	6.5
8	8.4	f	206	14	220	93.6	88	10.7
8	8.4	f	206	14	220	93.6	90.8	8.8
9	8.2	f	270	6	276	97.8	65.8	32.5
9	8.2	f	270	6	276	97.8	82.6	16.9
9	8.4	f	270	6	276	97.8	51.9	42.5
9	8.4	f	270	6	276	97.8	85.3	13.7
10	8.2	f	186	48	234	79.5	90.7	9.2
10	8.2	f	186	48	234	79.5	90.3	9.4
10	8.4	f	186	48	234	79.5	73.3	25.9
10	8.4	f	186	48	234	79.5	92.1	7.8

obili progre	Motili total	VCL	VSL	VAP	LIN	STR	WOB	ALH
1.8	17.9	26.1	14.3	19.0	54.8	75.3	72.9	2.2
0.4	6.1	27.5	9.0	15.6	32.6	57.5	56.6	1.3
0.1	5.4	26.6	3.6	10.4	13.6	34.7	39.3	0.9
0	3.1	23.1	2.5	8.4	10.9	30.2	36.2	0.0
1.7	15.3	32.6	18.5	23.5	56.8	78.8	72.2	2.6
0.1	8.3	26.7	8.4	14.8	31.5	56.9	55.3	0.9
0.2	4.9	24.3	7.0	13.5	28.7	51.6	55.6	0.7
0.1	4.6	31.2	5.6	12.3	18.0	45.6	39.5	1.0
0.2	5.6	24.2	6.8	12.6	28.2	54.2	52.1	1.4
0	3.6	23.2	6.0	10.4	26.1	58.2	44.8	0.0
0	3.8	24.6	3.9	10.6	15.8	36.9	42.9	0.0
0.1	7	24.5	4.9	12.0	20.2	41.3	49.0	1.1
0.5	7.9	24.8	11.1	15.5	44.7	71.4	62.7	1.5
0.5	14.6	22.4	8.5	12.0	37.9	70.9	53.4	1.1
0	2.9	16.4	2.7	5.6	16.2	47.6	34.0	0.0
0	23.8	25.0	4.6	9.8	18.4	47.1	39.1	0.0
0	4.7	21.0	7.1	11.6	33.7	60.8	55.3	0.0
0	2.9	18.5	2.9	6.6	15.5	43.5	35.7	0.0
0	4.6	19.0	3.5	6.4	18.1	53.9	33.7	0.0
0	2.4	19.2	1.2	6.1	6.0	18.9	32.0	0.0
2.2	26.3	30.4	17.1	23.5	56.4	73.0	77.2	2.3
0.7	15.7	26.8	13.2	18.8	49.1	69.8	70.4	2.3
0	5.3	25.8	4.5	11.4	17.5	39.8	44.0	0.0
0.1	7.9	25.5	6.7	12.7	26.4	53.2	49.6	2.0
1.9	15.8	26.0	12.0	16.6	45.9	72.0	63.8	1.3
0	1.2	17.2	3.6	5.0	20.8	71.1	29.2	0.0
0	3	12.0	1.4	2.2	11.4	62.6	18.1	0.0
0	3.7	10.8	3.3	4.0	31.0	84.3	36.8	0.0
2.4	29.1	29.7	13.9	19.9	46.7	69.7	66.9	3.0
0.4	26.7	24.6	10.9	16.4	44.3	66.7	66.4	1.5
0.2	7.4	21.8	7.5	12.2	34.4	61.3	56.0	0.8
0	4.9	18.0	4.1	9.1	23.0	45.4	50.6	0.0
1	12.9	22.3	10.4	14.8	46.5	70.3	66.2	1.4
0	1.8	6.9	0.4	0.4	6.0	100.0	6.0	0.0
0	5.3	21.4	4.6	10.8	21.5	42.6	50.5	0.0
0	4.4	26.9	5.8	11.6	21.4	49.4	43.3	0.0
0.7	13.1	25.9	10.3	16.1	39.6	64.0	61.9	1.5
0.2	7.4	20.2	9.2	12.5	45.5	73.6	61.8	0.5
0	5	20.1	3.6	8.5	17.8	42.3	42.1	0.0
0	4.5	18.6	6.9	10.6	37.1	65.1	57.1	0.0
0.2	8	24.4	6.9	13.1	28.1	52.4	53.6	0.4
0.5	5.2	26.7	9.2	14.4	34.6	64.1	54.0	1.6
0	6.1	23.2	5.5	11.6	23.8	47.7	49.8	0.0
0	9.2	21.9	7.3	12.1	33.5	60.4	55.5	0.0
2.3	22.1	25.3	12.5	17.1	49.3	73.0	67.5	0.5
0	21.3	13.4	3.0	5.3	22.2	56.3	39.4	0.0
0	5.5	20.2	6.1	10.0	30.0	61.0	49.2	0.0
0.9	13.6	24.3	8.7	14.6	35.9	59.4	60.4	1.1
0.2	5.3	27.6	9.5	15.6	34.4	60.8	56.5	1.5

0.5	10.5	29.7	11.8	18.6	39.6	63.4	62.4	1.2
0.4	13.2	23.2	9.7	14.4	41.8	67.6	61.9	1.1
0.2	13.4	24.9	10.6	15.7	42.6	67.4	63.2	0.6
2.8	43.1	29.0	14.4	20.8	49.7	69.4	71.7	2.3
5.4	56.2	32.3	17.2	24.2	53.4	71.2	75.0	2.4
6.7	49.8	33.0	19.7	25.7	59.7	76.6	77.9	2.8
5.1	43	32.3	18.9	25.0	58.5	75.7	77.3	2.6
0.2	3.6	23.3	6.2	11.1	26.4	55.3	47.7	1.2
0	3	24.0	5.1	11.0	21.1	46.2	45.7	0.0
0.2	5.9	27.1	7.3	13.0	26.9	56.2	47.8	1.7
0	5.6	28.5	5.7	13.2	19.9	42.9	46.4	0.0
0.3	8.5	27.8	10.7	16.4	38.5	65.4	59.0	2.6
0.3	11.7	24.0	9.0	13.4	37.5	67.0	56.0	3.1
2.1	20.9	30.0	15.7	21.1	52.4	74.5	70.4	1.9
4.3	44.2	33.2	16.4	23.2	49.4	70.6	70.0	2.7
1.3	29.5	27.7	10.9	17.4	39.3	62.7	62.7	2.7
0.5	21.7	27.8	8.7	15.3	31.4	56.9	55.2	2.0
2.5	34.7	28.1	14.4	19.7	51.0	72.8	70.1	2.4
6.4	54	32.6	19.0	24.6	58.3	77.1	75.5	2.9
16.4	81.8	39.8	23.7	30.9	59.6	76.8	77.6	2.6
3.7	30.3	30.0	17.4	22.3	58.0	77.8	74.6	2.1
7.8	49.9	33.1	21.6	26.4	65.3	81.9	79.8	2.3
4.6	38	31.5	20.5	25.1	65.1	81.8	79.7	2.3
4.8	39.7	29.4	17.0	22.1	58.0	77.3	75.0	2.3
2.2	49.4	28.7	13.5	19.8	47.1	68.3	69.0	2.7
17.4	65.1	46.5	33.3	38.5	71.5	86.3	82.8	2.6
3.6	59.6	28.3	17.7	22.7	62.6	78.0	80.2	2.2
0	6.4	25.4	6.3	12.4	24.8	50.6	48.9	0.0
0.2	6.7	30.3	7.1	14.1	23.4	50.2	46.6	0.7
1.2	11.9	25.1	12.3	17.4	48.9	70.5	69.4	2.2
0.4	9.2	24.4	7.9	14.0	32.2	56.0	57.5	0.9
1.6	34.1	28.8	12.6	18.6	43.8	67.8	64.6	2.1
0.5	17.4	25.5	10.3	16.2	40.4	63.8	63.4	1.0
5.6	48.1	34.5	19.9	26.7	57.6	74.5	77.3	1.9
0.9	14.6	26.3	10.7	16.6	40.6	64.2	63.1	2.4
0.1	9.3	26.9	6.2	12.3	23.2	50.8	45.7	1.9
0.3	9.7	29.2	9.2	17.0	31.7	54.3	58.3	3.4
0.8	26.7	28.1	9.8	17.4	35.0	56.4	62.0	3.1
0.1	7.9	25.9	7.5	14.7	28.9	50.9	56.7	1.4

BCF
7.3
5.2
0.4
0.0
6.8
0.0
3.7
1.7
4.0
0.0
0.0
0.0
6.6
1.6
0.0
0.0
0.0
0.0
0.0
0.0
9.1
8.4
0.0
0.0
6.0
0.0
0.0
0.0
5.8
5.3
0.0
0.0
4.5
0.0
0.0
0.0
3.2
2.7
0.0
0.0
1.0
8.7
0.0
0.0
2.3
0.0
0.0
1.9
4.0

4.6
3.3
4.0
6.9
7.6
7.1
7.6
3.5
0.0
1.3
0.0
9.1
6.3
9.4
8.4
7.4
8.1
6.6
6.8
7.0
7.5
8.4
7.8
6.9
6.8
8.4
7.7
0.0
4.5
8.4
3.2
6.9
4.2
7.4
5.3
1.0
2.6
4.6
0.5
