# Supplementary material for: Assessment of Sperm Viability and Computer-Assisted Motility Analysis in Budgerigars (Melopsittacus undulatus): Effect of Several In Vitro Processing Conditions
Source: Vet Med Int. 2022 Mar 22;2022:5997320. doi: 10.1155/2022/5997320 (PMC8964162; doi:10.1155/2022/5997320)
Supplement: Supplementary Materials — The data are provided in the Supplementary Materials. [file 5997320.f1.zip › 5997320.f1/dataset_protocollo_2_pappagalli.pdf]

pool	Diluizione	TF	Vivi	Morti	TOT	Vitalità
1	3	25	157	61	218	72.0
1	3	4	157	61	218	72.0
1	4	25	157	61	218	72.0
1	4	4	157	61	218	72.0
2	3	25	178	28	206	86.4
2	3	4	178	28	206	86.4
2	4	25	178	28	206	86.4
2	4	4	178	28	206	86.4
3	3	25	183	30	213	85.9
3	3	4	183	30	213	85.9
3	4	25	183	30	213	85.9
3	4	4	183	30	213	85.9
4	3	25	166	38	204	81.4
4	3	4	166	38	204	81.4
4	4	25	166	38	204	81.4
4	4	4	166	38	204	81.4
5	3	25	307	27	334	91.9
5	3	4	307	27	334	91.9
5	4	25	307	27	334	91.9
5	4	4	307	27	334	91.9
6	3	25	243	9	252	96.4
6	3	4	243	9	252	96.4
6	4	25	243	9	252	96.4
6	4	4	243	9	252	96.4
7	3	25	194	7	201	96.5
7	3	4	194	7	201	96.5
7	4	25	194	7	201	96.5
7	4	4	194	7	201	96.5
1	3	25	169	34	203	83.3
1	3	38	169	34	203	83.3
1	3	25	169	34	203	83.3
1	3	38	169	34	203	83.3
2	3	25	174	39	213	81.7
2	3	38	174	39	213	81.7
2	3	25	174	39	213	81.7
2	3	38	174	39	213	81.7
3	3	25	171	66	237	72.2
3	3	38	171	66	237	72.2
3	3	25	171	66	237	72.2
3	3	38	171	66	237	72.2
4	3	25	169	38	207	81.6
4	3	38	169	38	207	81.6
4	3	25	169	38	207	81.6
4	3	38	169	38	207	81.6
5	3	25	218	8	226	96.5
5	3	38	218	8	226	96.5
5	3	25	218	8	226	96.5
5	3	38	218	8	226	96.5
6	3	25	194	6	200	97.0

6	3	38	194	6	200	97.0
6	3	25	194	6	200	97.0
6	3	38	194	6	200	97.0
7	3	25	206	12	218	94.5
7	3	38	206	12	218	94.5
7	3	25	206	12	218	94.5
7	3	38	206	12	218	94.5
8	3	25	206	14	220	93.6
8	3	38	206	14	220	93.6
8	3	25	206	14	220	93.6
8	3	38	206	14	220	93.6
9	3	25	270	6	276	97.8
9	3	38	270	6	276	97.8
9	3	25	270	6	276	97.8
9	3	38	270	6	276	97.8
10	3	25	186	48	234	79.5
10	3	38	186	48	234	79.5
10	3	25	186	48	234	79.5
10	3	38	186	48	234	79.5

Statici	bili non progre	Mobili progres	Motili totali	VCL	VSL	VAP
57.6	32.6	9.8	42.4	40.8	25.6	31.9
64.3	30.7	5	35.7	35.3	19.9	27.0
82.5	13.7	3.8	17.5	40.3	27.2	32.5
61.8	32.2	6	38.2	37.3	20.3	27.4
78.5	19.5	1.9	21.4	29.5	15.2	20.7
79.7	16.2	4	20.2	41.6	21.8	28.6
81.5	16.8	1.8	18.6	28.8	15.3	20.8
68.9	28.8	2.4	31.2	29.9	12.6	18.1
85.3	13.3	1.4	14.7	27.8	17.3	21.6
84.8	13.7	1.4	15.1	28.9	12.7	18.3
83.1	14	2.8	16.8	39.3	22.4	29.8
87.9	9.3	2.7	12	33.5	20.8	25.0
88.3	11	0.7	11.7	25.3	10.4	16.3
87.9	12.1	0	12.1	24.8	7.5	13.4
83.6	15.3	1.2	16.5	25.7	13.9	18.2
78	20.9	1.1	22	27.2	12.9	18.1
48.1	38	13.9	51.9	43.6	31.6	36.5
49.9	33.5	16.6	50.1	52.3	38.4	44.4
50.9	36.8	12.3	49.1	41.5	28.1	33.7
75.8	21.9	2.4	24.3	30.3	15.4	21.8
42.2	45.9	12	57.9	40.3	24.6	31.4
38.9	48.3	12.8	61.1	38.3	23.4	29.5
65.6	29.1	5.2	34.3	32.2	20.7	25.5
53.4	40.3	6.3	46.6	31.4	17.6	23.1
65.2	30.6	4.2	34.8	31.3	15.8	21.7
59.1	34.8	6	40.8	33.9	17.9	24.6
73.9	19.7	6.5	26.2	44.8	30.4	35.6
59.2	31.8	9	40.8	42.0	25.7	32.3
94.7	5.1	0.2	5.3	27.6	9.5	15.6
89.5	10	0.5	10.5	29.7	11.8	18.6
86.8	12.8	0.4	13.2	23.2	9.7	14.4
86.7	13.2	0.2	13.4	24.9	10.6	15.7
56.9	40.3	2.8	43.1	29.0	14.4	20.8
43.8	50.8	5.4	56.2	32.3	17.2	24.2
50.2	43.1	6.7	49.8	33.0	19.7	25.7
57	37.9	5.1	43	32.3	18.9	25.0
96.4	3.4	0.2	3.6	23.3	6.2	11.1
97	3	0	3	24.0	5.1	11.0
94.1	5.7	0.2	5.9	27.1	7.3	13.0
94.4	5.6	0	5.6	28.5	5.7	13.2
91.5	8.2	0.3	8.5	27.8	10.7	16.4
88.3	11.4	0.3	11.7	24.0	9.0	13.4
79.1	18.8	2.1	20.9	30.0	15.7	21.1
55.8	39.9	4.3	44.2	33.2	16.4	23.2
70.5	28.2	1.3	29.5	27.7	10.9	17.4
78.3	21.2	0.5	21.7	27.8	8.7	15.3
65.3	32.2	2.5	34.7	28.1	14.4	19.7
46	47.6	6.4	54	32.6	19.0	24.6
18.2	65.4	16.4	81.8	39.8	23.7	30.9

69.7	26.6	3.7	30.3	30.0	17.4	22.3
50.1	42.1	7.8	49.9	33.1	21.6	26.4
62	33.4	4.6	38	31.5	20.5	25.1
60.3	34.9	4.8	39.7	29.4	17.0	22.1
50.6	47.2	2.2	49.4	28.7	13.5	19.8
34.9	47.7	17.4	65.1	46.5	33.3	38.5
40.4	56	3.6	59.6	28.3	17.7	22.7
93.6	6.4	0	6.4	25.4	6.3	12.4
93.3	6.5	0.2	6.7	30.3	7.1	14.1
88	10.7	1.2	11.9	25.1	12.3	17.4
90.8	8.8	0.4	9.2	24.4	7.9	14.0
65.8	32.5	1.6	34.1	28.8	12.6	18.6
82.6	16.9	0.5	17.4	25.5	10.3	16.2
51.9	42.5	5.6	48.1	34.5	19.9	26.7
85.3	13.7	0.9	14.6	26.3	10.7	16.6
90.7	9.2	0.1	9.3	26.9	6.2	12.3
90.3	9.4	0.3	9.7	29.2	9.2	17.0
73.3	25.9	0.8	26.7	28.1	9.8	17.4
92.1	7.8	0.1	7.9	25.9	7.5	14.7

LIN	STR	WOB	ALH	BCF
62.6	80.2	78.2	2.3	8.1
56.4	73.8	76.4	2.8	7.7
67.6	84.0	80.5	2.0	9.2
54.4	73.9	73.6	2.6	7.1
51.6	73.4	70.3	2.1	7.8
52.4	76.3	68.7	2.2	8.6
53.0	73.4	72.2	2.3	5.9
42.3	69.7	60.7	3.3	6.7
62.3	80.2	77.6	2.5	7.8
43.9	69.2	63.4	3.3	5.6
56.9	75.0	75.9	2.3	7.5
62.0	82.9	74.7	1.7	7.4
41.0	63.9	64.3	2.7	6.1
30.1	55.8	53.9	0.0	0.0
54.3	76.6	70.9	2.4	7.3
47.4	71.0	66.7	2.1	5.4
72.5	86.5	83.8	1.9	8.5
73.5	86.5	85.0	2.1	8.2
67.7	83.4	81.2	2.4	8.7
50.6	70.4	71.8	3.1	4.8
61.1	78.3	78.0	2.8	7.7
61.2	79.4	77.1	2.6	7.8
64.3	81.0	79.3	2.5	8.0
56.1	76.5	73.3	2.3	8.7
50.7	73.1	69.3	2.1	8.3
52.8	72.7	72.6	2.3	7.6
67.8	85.2	79.5	2.1	8.8
61.3	79.6	77.0	2.4	7.8
34.4	60.8	56.5	1.5	4.0
39.6	63.4	62.4	1.2	4.6
41.8	67.6	61.9	1.1	3.3
42.6	67.4	63.2	0.6	4.0
49.7	69.4	71.7	2.3	6.9
53.4	71.2	75.0	2.4	7.6
59.7	76.6	77.9	2.8	7.1
58.5	75.7	77.3	2.6	7.6
26.4	55.3	47.7	1.2	3.5
21.1	46.2	45.7	0.0	0.0
26.9	56.2	47.8	1.7	1.3
19.9	42.9	46.4	0.0	0.0
38.5	65.4	59.0	2.6	9.1
37.5	67.0	56.0	3.1	6.3
52.4	74.5	70.4	1.9	9.4
49.4	70.6	70.0	2.7	8.4
39.3	62.7	62.7	2.7	7.4
31.4	56.9	55.2	2.0	8.1
51.0	72.8	70.1	2.4	6.6
58.3	77.1	75.5	2.9	6.8
59.6	76.8	77.6	2.6	7.0

58.0	77.8	74.6	2.1	7.5
65.3	81.9	79.8	2.3	8.4
65.1	81.8	79.7	2.3	7.8
58.0	77.3	75.0	2.3	6.9
47.1	68.3	69.0	2.7	6.8
71.5	86.3	82.8	2.6	8.4
62.6	78.0	80.2	2.2	7.7
24.8	50.6	48.9	0.0	0.0
23.4	50.2	46.6	0.7	4.5
48.9	70.5	69.4	2.2	8.4
32.2	56.0	57.5	0.9	3.2
43.8	67.8	64.6	2.1	6.9
40.4	63.8	63.4	1.0	4.2
57.6	74.5	77.3	1.9	7.4
40.6	64.2	63.1	2.4	5.3
23.2	50.8	45.7	1.9	1.0
31.7	54.3	58.3	3.4	2.6
35.0	56.4	62.0	3.1	4.6
28.9	50.9	56.7	1.4	0.5
