# Supplementary material for: Assessment of Sperm Viability and Computer-Assisted Motility Analysis in Budgerigars (Melopsittacus undulatus): Effect of Several In Vitro Processing Conditions
Source: Vet Med Int. 2022 Mar 22;2022:5997320. doi: 10.1155/2022/5997320 (PMC8964162; doi:10.1155/2022/5997320)
Supplement: Supplementary Materials — The data are provided in the Supplementary Materials. [file 5997320.f1.zip › 5997320.f1/dataset_protocollo_3_pappagalli.pdf]

individuo	Temperatura	Statici	Mobili non progressivi	Mobili progressivi	Motili totali
1	38	81.4	17.9	0.7	18.6
2	38	87.9	12.1	0	12.1
3	38	71.1	26.7	2.2	28.9
4	38	90.6	8.3	1.1	9.4
5	38	68.7	28.1	3.3	31.4
6	38	94.6	5.4	0	5.4
7	38	84	15	1	16
8	38	88.7	11.3	0	11.3
9	38	86.6	13.4	0	13.4
1	41	46	51.3	2.8	54.1
2	41	67.7	30.7	1.6	32.3
3	41	58.2	40.2	1.6	41.8
4	41	48.8	40.4	10.8	51.2
5	41	39	47.5	13.5	61
6	41	84	16	0	16
7	41	72.3	24.7	3	27.7
8	41	90.8	9.2	0	9.2
9	41	66.9	29.3	3.8	33.1

VCL	VSL	VAP	LIN	STR	WOB	ALH	BCF
25.2	7.5	13.2	30.0	57.0	52.5	2.2	5.5
21.9	11.1	15.7	50.5	70.5	71.7	0.0	0.0
28.9	15.6	21.3	53.9	73.2	73.6	2.5	5.1
21.2	10.9	14.3	51.3	76.1	67.5	0.8	5.4
29.8	16.5	22.5	55.5	73.5	75.4	2.2	6.9
26.5	13.1	19.0	49.5	69.2	71.5	0.0	0.0
25.6	12.9	18.1	50.4	71.1	70.8	2.0	6.5
26.1	12.0	19.0	46.0	63.3	72.7	0.0	0.0
28.4	12.5	18.1	44.2	69.3	63.8	0.0	0.0
28.4	15.2	21.0	53.5	72.2	74.0	2.4	7.3
26.1	13.2	18.5	50.7	71.6	70.9	1.9	8.4
25.2	10.6	16.0	41.9	66.1	63.4	2.0	6.5
40.1	24.0	30.5	59.8	78.8	76.0	2.4	9.9
45.2	30.8	37.4	68.1	82.3	82.8	2.4	7.6
21.3	10.1	14.7	47.4	68.6	69.1	0.0	0.0
29.7	17.9	22.9	60.5	78.3	77.3	1.6	9.9
24.2	8.6	14.0	35.6	61.6	57.8	0.0	0.0
33.3	20.6	26.3	62.0	78.6	78.9	1.8	7.1
